# Supplementary material for: A Critical View of the Application of the APEX Software (Aqueous Photochemistry of Environmentally-Occurring Xenobiotics) to Predict Photoreaction Kinetics in Surface Freshwaters
Source: Molecules. 2019 Dec 18;25(1):9. doi: 10.3390/molecules25010009 (PMC7017383; doi:10.3390/molecules25010009)
Supplement: Supplementary file 1 [file molecules-25-00009-s001.zip › 3.2.4_gcc-4.4.0.zip!/3.2.4_gcc-4.4.0/3.2.4_gcc-4.4.0/doc/octave/html/interpreter/Octave-Sources-_0028m_002dfiles_0029.html]

Octave Sources (m-files) - Untitled


Next: C++ Sources,
Previous: General Guidelines,
Up: Contributing Guidelines


---

### D.3 Octave Sources (m-files)

Don't use tabs. Tabs cause trouble. If you are used to them, set up your editor
so that it converts tabs to spaces. Indent the bodies of the statement blocks.
Recommended indent is 2 spaces. When calling functions, put spaces after commas
and before the calling parentheses, like this:

```
       x = max (sin (y+3), 2);
```

An exception are matrix and vector constructors:

```
       [sin(x), cos(x)]
```

Here, putting spaces after `sin`, `cos` would result in a parse error.
In indexing expression, do not put a space after the identifier (this
differentiates indexing and function calls nicely). The space after comma is not
necessary if index expressions are simple, i.e., you may write

```
       A(:,i,j)
```

but

```
       A([1:i-1;i+1:n], XI(:,2:n-1))
```

Use lowercase names if possible. Uppercase is acceptable for variable names
consisting of 1-2 letters. Do not use mixed case names. Function names must be
lowercase. Function names are global, so choose them wisely.

Always use a specific end-of-block statement (like `endif`,
`endswitch`) rather than generic `end`. Enclose the `if`,
`while`, `until` and `switch` conditions in parentheses,
like in C:

```
     if (isvector (a))
       s = sum(a);
     endif
```

Do not do this, however, with `for`:

```
     for i = 1:n
       b(i) = sum (a(:,i));
     endfor
```
